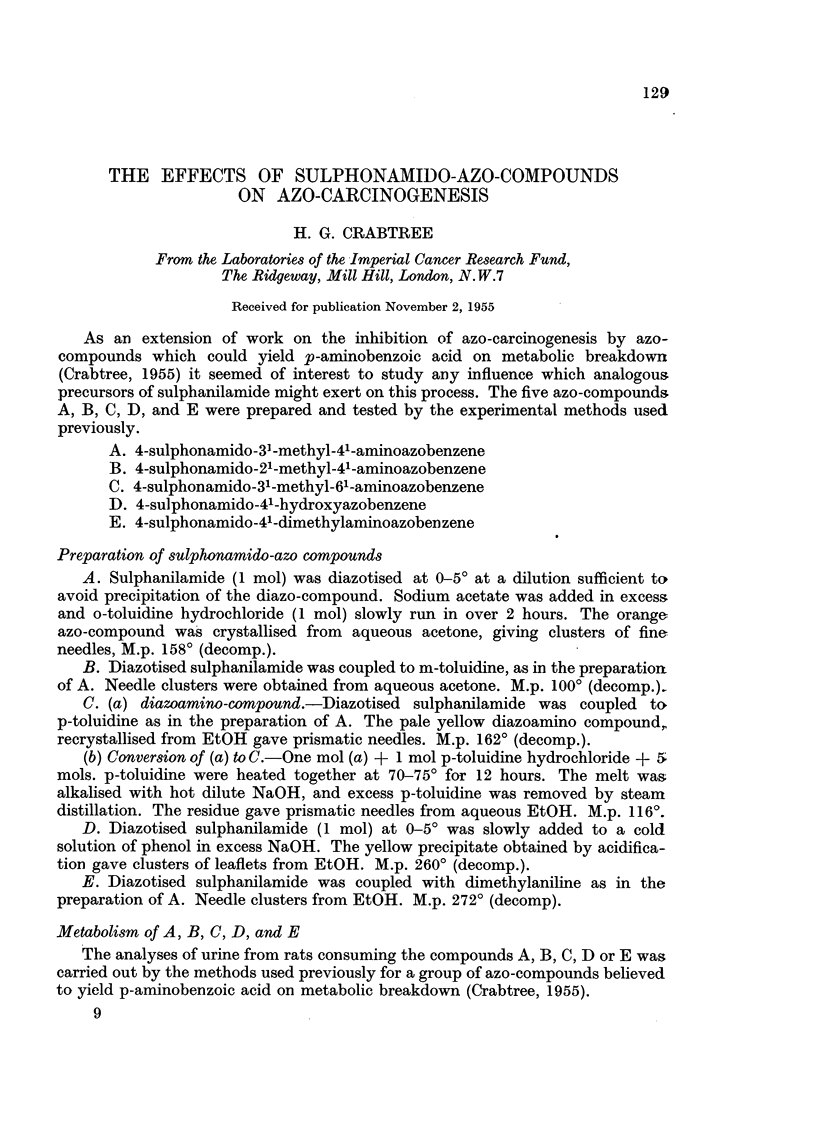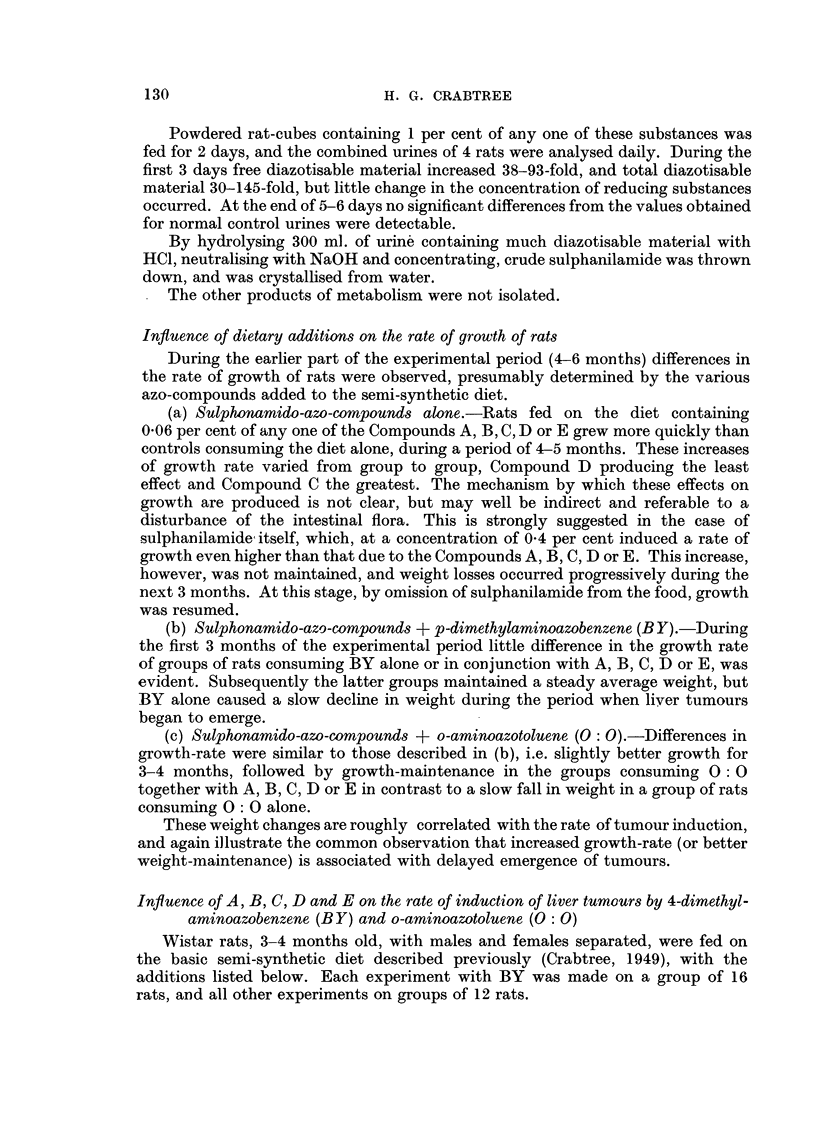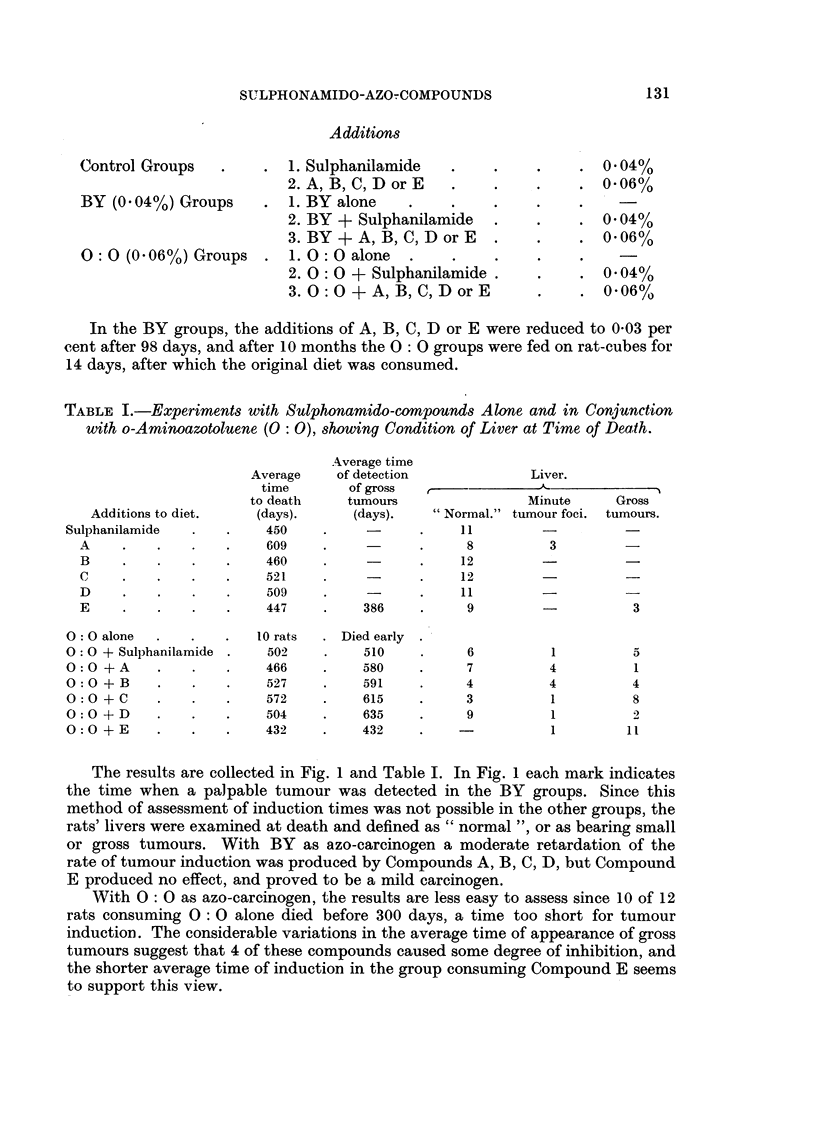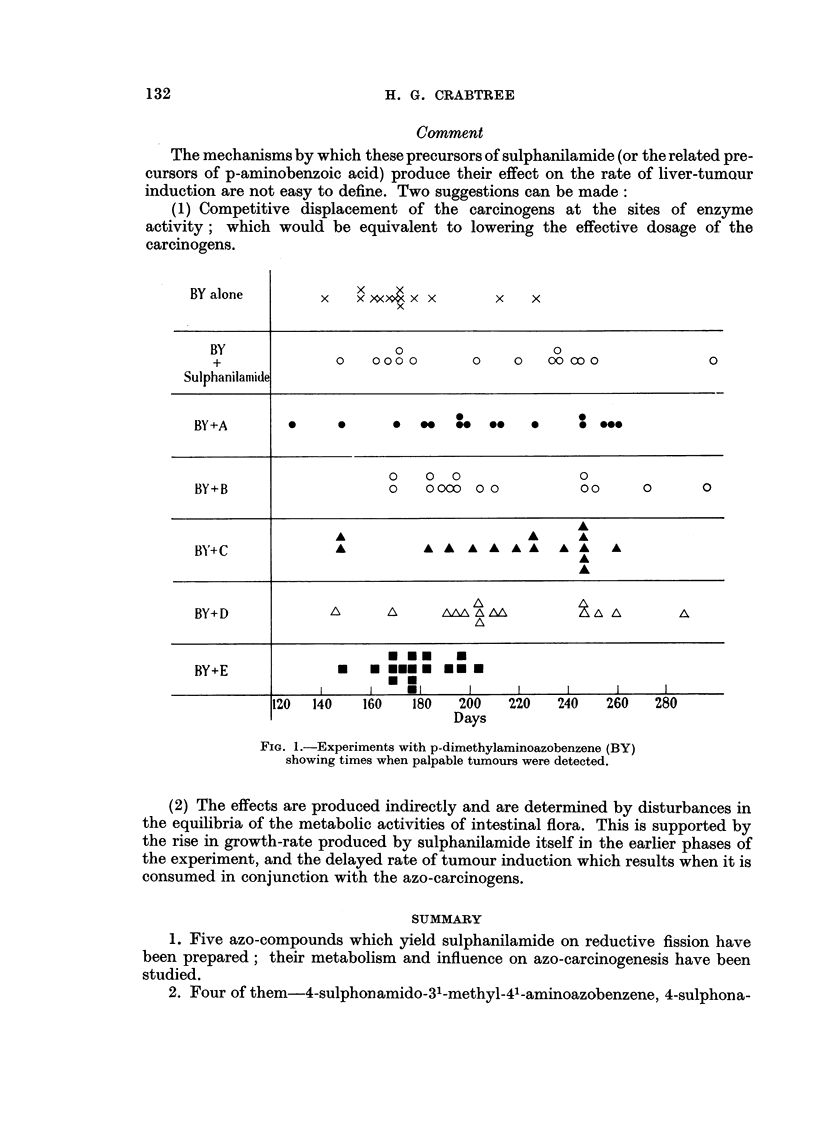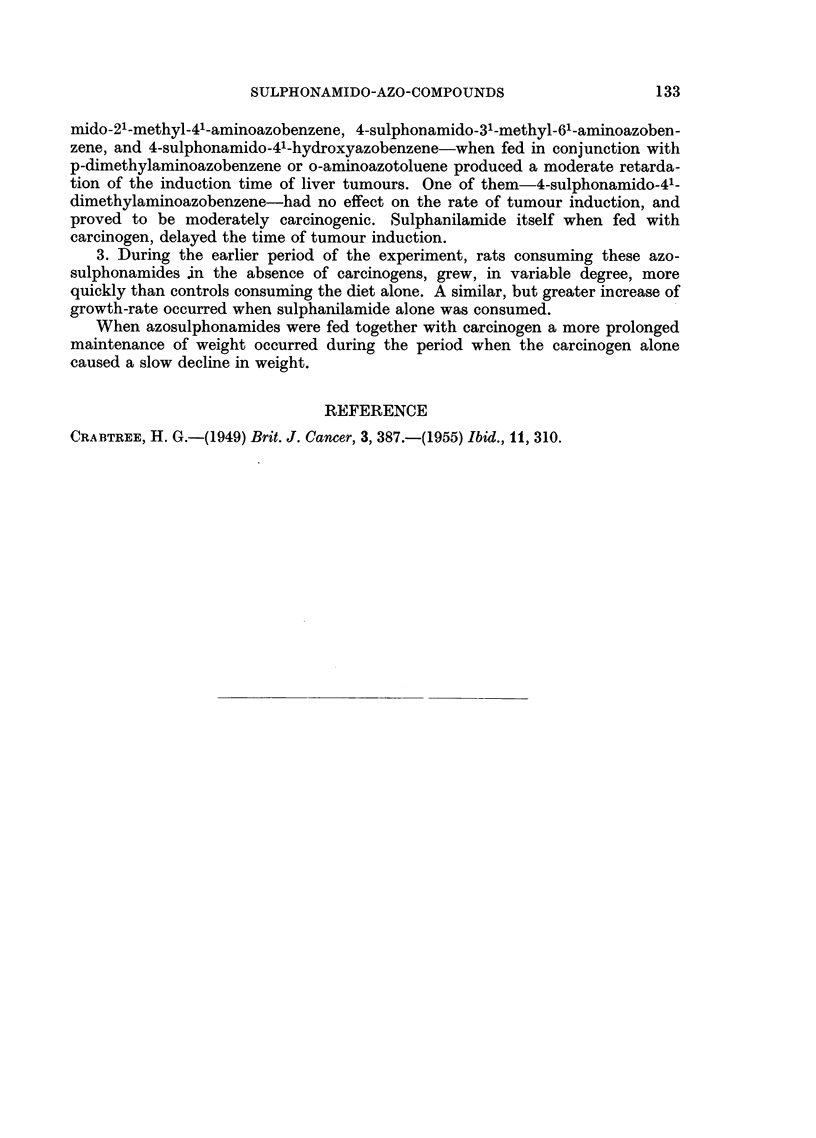# The Effects of Sulphonamido-Azo-Compounds on Azo-Carcinogenesis

**DOI:** 10.1038/bjc.1956.17

**Published:** 1956-03

**Authors:** H. G. Crabtree


### THE EFFECTS OF SULPHONAMIDO-AZO-COMPOUNDS ON AZO-CARCINOGENESIS

H. G. CRABTREE

*From the Laboratories of the Imperial Cancer Research Fund, The Ridgeway, Mill Hill, London, N.W.7*

Received for publication November 2, 1955

As an extension of work on the inhibition of azo-carcinogenesis by azo-compounds which could yield *p*-aminobenzoic acid on metabolic breakdown (Crabtree, 1955) it seemed of interest to study any influence which analogous precursors of sulphanilamide might exert on this process. The five azo-compounds A, B, C, D, and E were prepared and tested by the experimental methods used previously.

A. 4-sulphonamido-$3^1$-methyl-$4^1$-aminoazobenzene
B. 4-sulphonamido-$2^1$-methyl-$4^1$-aminoazobenzene
C. 4-sulphonamido-$3^1$-methyl-$6^1$-aminoazobenzene
D. 4-sulphonamido-$4^1$-hydroxyazobenzene
E. 4-sulphonamido-$4^1$-dimethylaminoazobenzene

*Preparation of sulphonamido-azo compounds*

*A*. Sulphanilamide (1 mol) was diazotised at 0–5° at a dilution sufficient to avoid precipitation of the diazo-compound. Sodium acetate was added in excess and o-toluidine hydrochloride (1 mol) slowly run in over 2 hours. The orange azo-compound was crystallised from aqueous acetone, giving clusters of fine needles, M.p. 158° (decomp.).

*B*. Diazotised sulphanilamide was coupled to m-toluidine, as in the preparation of A. Needle clusters were obtained from aqueous acetone. M.p. 100° (decomp.).

*C*. (*a*) *diazoamino-compound*.—Diazotised sulphanilamide was coupled to p-toluidine as in the preparation of A. The pale yellow diazoamino compound, recrystallised from EtOH gave prismatic needles. M.p. 162° (decomp.).

(*b*) *Conversion of* (*a*) *to C*.—One mol (*a*) + 1 mol p-toluidine hydrochloride + 5 mols. p-toluidine were heated together at 70–75° for 12 hours. The melt was alkalised with hot dilute NaOH, and excess p-toluidine was removed by steam distillation. The residue gave prismatic needles from aqueous EtOH. M.p. 116°.

*D*. Diazotised sulphanilamide (1 mol) at 0–5° was slowly added to a cold solution of phenol in excess NaOH. The yellow precipitate obtained by acidification gave clusters of leaflets from EtOH. M.p. 260° (decomp.).

*E*. Diazotised sulphanilamide was coupled with dimethylaniline as in the preparation of A. Needle clusters from EtOH. M.p. 272° (decomp).

*Metabolism of A, B, C, D, and E*

The analyses of urine from rats consuming the compounds A, B, C, D or E was carried out by the methods used previously for a group of azo-compounds believed to yield p-aminobenzoic acid on metabolic breakdown (Crabtree, 1955).

Powdered rat-cubes containing 1 per cent of any one of these substances was fed for 2 days, and the combined urines of 4 rats were analysed daily. During the first 3 days free diazotisable material increased 38–93-fold, and total diazotisable material 30–145-fold, but little change in the concentration of reducing substances occurred. At the end of 5–6 days no significant differences from the values obtained for normal control urines were detectable.

By hydrolysing 300 ml. of urine containing much diazotisable material with HCl, neutralising with NaOH and concentrating, crude sulphanilamide was thrown down, and was crystallised from water.

The other products of metabolism were not isolated.

*Influence of dietary additions on the rate of growth of rats*

During the earlier part of the experimental period (4–6 months) differences in the rate of growth of rats were observed, presumably determined by the various azo-compounds added to the semi-synthetic diet.

(a) *Sulphonamido-azo-compounds alone.*—Rats fed on the diet containing 0·06 per cent of any one of the Compounds A, B, C, D or E grew more quickly than controls consuming the diet alone, during a period of 4–5 months. These increases of growth rate varied from group to group, Compound D producing the least effect and Compound C the greatest. The mechanism by which these effects on growth are produced is not clear, but may well be indirect and referable to a disturbance of the intestinal flora. This is strongly suggested in the case of sulphanilamide itself, which, at a concentration of 0·4 per cent induced a rate of growth even higher than that due to the Compounds A, B, C, D or E. This increase, however, was not maintained, and weight losses occurred progressively during the next 3 months. At this stage, by omission of sulphanilamide from the food, growth was resumed.

(b) *Sulphonamido-azo-compounds + p-dimethylaminoazobenzene (BY).*—During the first 3 months of the experimental period little difference in the growth rate of groups of rats consuming BY alone or in conjunction with A, B, C, D or E, was evident. Subsequently the latter groups maintained a steady average weight, but BY alone caused a slow decline in weight during the period when liver tumours began to emerge.

(c) *Sulphonamido-azo-compounds + o-aminoazotoluene (O : O).*—Differences in growth-rate were similar to those described in (b), i.e. slightly better growth for 3–4 months, followed by growth-maintenance in the groups consuming O : O together with A, B, C, D or E in contrast to a slow fall in weight in a group of rats consuming O : O alone.

These weight changes are roughly correlated with the rate of tumour induction, and again illustrate the common observation that increased growth-rate (or better weight-maintenance) is associated with delayed emergence of tumours.

*Influence of A, B, C, D and E on the rate of induction of liver tumours by 4-dimethylaminoazobenzene (BY) and o-aminoazotoluene (O : O)*

Wistar rats, 3–4 months old, with males and females separated, were fed on the basic semi-synthetic diet described previously (Crabtree, 1949), with the additions listed below. Each experiment with BY was made on a group of 16 rats, and all other experiments on groups of 12 rats.

*Additions*

| | | |
|---|---|---|
| Control Groups | 1. Sulphanilamide | 0·04% |
| | 2. A, B, C, D or E | 0·06% |
| BY (0·04%) Groups | 1. BY alone | — |
| | 2. BY + Sulphanilamide | 0·04% |
| | 3. BY + A, B, C, D or E | 0·06% |
| O : O (0·06%) Groups | 1. O : O alone | — |
| | 2. O : O + Sulphanilamide | 0·04% |
| | 3. O : O + A, B, C, D or E | 0·06% |

In the BY groups, the additions of A, B, C, D or E were reduced to 0·03 per cent after 98 days, and after 10 months the O : O groups were fed on rat-cubes for 14 days, after which the original diet was consumed.

TABLE I.—*Experiments with Sulphonamido-compounds Alone and in Conjunction with o-Aminoazotoluene (O : O), showing Condition of Liver at Time of Death.*

| Additions to diet. | Average time to death (days). | Average time of detection of gross tumours (days). | Liver. "Normal." | Minute tumour foci. | Gross tumours. |
|---|---|---|---|---|---|
| Sulphanilamide | 450 | — | 11 | — | — |
| A | 609 | — | 8 | 3 | — |
| B | 460 | — | 12 | — | — |
| C | 521 | — | 12 | — | — |
| D | 509 | — | 11 | — | — |
| E | 447 | 386 | 9 | — | 3 |
| O : O alone | 10 rats | Died early | | | |
| O : O + Sulphanilamide | 502 | 510 | 6 | 1 | 5 |
| O : O + A | 466 | 580 | 7 | 4 | 1 |
| O : O + B | 527 | 591 | 4 | 4 | 4 |
| O : O + C | 572 | 615 | 3 | 1 | 8 |
| O : O + D | 504 | 635 | 9 | 1 | 2 |
| O : O + E | 432 | 432 | — | 1 | 11 |

The results are collected in Fig. 1 and Table I. In Fig. 1 each mark indicates the time when a palpable tumour was detected in the BY groups. Since this method of assessment of induction times was not possible in the other groups, the rats' livers were examined at death and defined as " normal ", or as bearing small or gross tumours. With BY as azo-carcinogen a moderate retardation of the rate of tumour induction was produced by Compounds A, B, C, D, but Compound E produced no effect, and proved to be a mild carcinogen.

With O : O as azo-carcinogen, the results are less easy to assess since 10 of 12 rats consuming O : O alone died before 300 days, a time too short for tumour induction. The considerable variations in the average time of appearance of gross tumours suggest that 4 of these compounds caused some degree of inhibition, and the shorter average time of induction in the group consuming Compound E seems to support this view.

*Comment*

The mechanisms by which these precursors of sulphanilamide (or the related precursors of p-aminobenzoic acid) produce their effect on the rate of liver-tumour induction are not easy to define. Two suggestions can be made :

(1) Competitive displacement of the carcinogens at the sites of enzyme activity ; which would be equivalent to lowering the effective dosage of the carcinogens.

FIG. 1.—Experiments with p-dimethylaminoazobenzene (BY) showing times when palpable tumours were detected.

(2) The effects are produced indirectly and are determined by disturbances in the equilibria of the metabolic activities of intestinal flora. This is supported by the rise in growth-rate produced by sulphanilamide itself in the earlier phases of the experiment, and the delayed rate of tumour induction which results when it is consumed in conjunction with the azo-carcinogens.

SUMMARY

1. Five azo-compounds which yield sulphanilamide on reductive fission have been prepared ; their metabolism and influence on azo-carcinogenesis have been studied.

2. Four of them—4-sulphonamido-$3^1$-methyl-$4^1$-aminoazobenzene, 4-sulphonamido-2[1]-methyl-4[1]-aminoazobenzene, 4-sulphonamido-3[1]-methyl-6[1]-aminoazobenzene, and 4-sulphonamido-4[1]-hydroxyazobenzene—when fed in conjunction with p-dimethylaminoazobenzene or o-aminoazotoluene produced a moderate retardation of the induction time of liver tumours. One of them—4-sulphonamido-4[1]-dimethylaminoazobenzene—had no effect on the rate of tumour induction, and proved to be moderately carcinogenic. Sulphanilamide itself when fed with carcinogen, delayed the time of tumour induction.

3. During the earlier period of the experiment, rats consuming these azosulphonamides in the absence of carcinogens, grew, in variable degree, more quickly than controls consuming the diet alone. A similar, but greater increase of growth-rate occurred when sulphanilamide alone was consumed.

When azosulphonamides were fed together with carcinogen a more prolonged maintenance of weight occurred during the period when the carcinogen alone caused a slow decline in weight.

### REFERENCE

Crabtree, H. G.—(1949) *Brit. J. Cancer*, **3**, 387.—(1955) *Ibid.*, **11**, 310.